# 

**Published:** 1977

**Authors:** 


### Contents

3 **Who Cares for Children ?**
S. D. M. Court

9 **Forty Years of Obstetrics in General Practice**
Christopher Morgans

